# One adaptor, multiple outcomes: decoding TRIFosome heterogeneity

**DOI:** 10.1042/BST20260704

**Published:** 2026-10-01

**Authors:** Prasanna Suresh, Yu P. Zhang, Clare Bryant, David Klenerman

**Affiliations:** 1 Yusuf Hamied Department of Chemistry, University of Cambridge, Cambridge, U.K.; 2 Department of Veterinary Medicine, University of Cambridge, Cambridge, U.K.; 3 Department of Medicine, University of Cambridge, Cambridge, U.K.

**Keywords:** inflammation, innate immunity, SMOC, toll-like receptors, TRIF, TRIFosome

## Abstract

Innate immune signalling is uniquely driven through the formation of supramolecular organising centres (SMOCs), which couple receptor activation with downstream effector recruitment. The adaptor protein TRIF coordinates interferon, inflammatory, and cell death responses downstream of TLR3 and TLR4 activation, yet exactly how this protein drives such diverse outputs remains unresolved. Recent structural studies of recombinant TRIF, alongside single-molecule fluorescence imaging of endogenously expressed TRIF in live cells, reveal that TRIF assembles into complexes with heterogeneous size and morphology, with monomeric and low-order complexes associated with early-phase interferon signalling and mature, RIPK1-containing complexes associated with NF-κB activation. This review explores how differences in the structural architecture of the TRIFosome, from TIR-mediated oligomerisation through to RHIM-mediated cross-linking, may underlie this divergence in signalling outcome. Finally, this review discusses the translational opportunities that coordinating scaffold assembly with downstream function presents, including structure-selective therapeutics capable of shifting the balance between TRIF’s signalling outputs and novel diagnostics that could be built on TRIFosome heterogeneity as a molecular fingerprint of disease.

## Introduction

Classical signal transduction relies on the generation of secondary messengers (such as cAMP or calcium ions in G-protein-coupled receptor signalling) or linear enzymatic cascades (such as the MAPK cascade downstream of receptor tyrosine kinases). In stark contrast, innate immune activation is uniquely characterised by the stimulus-driven assembly of supramolecular organising centres (SMOCs) [1]. Upon detection of pathogen-associated molecular patterns (PAMPs), pattern recognition receptors nucleate the assembly of multimeric heteroprotein complexes. SMOCs utilise domain-domain interactions to build stable, multivalent scaffolds that facilitate the recruitment of downstream ubiquitin ligases and kinases [2]. This localised spatial clustering of adaptor proteins and downstream effectors enables the dual-mode action of SMOCs as both a threshold and an amplifier, enabling cells to respond to small levels of potent agonists to mount a robust defence against infection, whilst mitigating the risk of aberrant autoinflammatory activation [1].

Within Toll-like receptor (TLR) signalling, this SMOC-driven architecture bifurcates into two primary cascades defined by their respective adaptor proteins: MyD88 and TRIF [3]. The MyD88-dependent pathway, downstream of the IL-1 receptor family and all human TLRs with the exception of TLR3, and regulated via the formation of the MyDDosome signalling complex, is hypothesised to drive predominantly inflammatory responses [4,5]. This contrasts with TRIF signalling, which is initiated downstream of only TLR3 and TLR4, and is hypothesised to coordinate a combination of inflammatory signalling, interferon, and cell death responses [6,7]. Both these proteins are capable of self-assembling into filaments *in vitro*, facilitated through Toll/interleukin-1 receptor/resistance protein (TIR) domain interactions [8,9]. Structural studies of purified MyD88 and TRIF have been invaluable for understanding their possible modes of self-association and interactions with other adaptor proteins, such as Mal/TIRAP and TRAM, respectively. However, these models have limitations when it comes to capturing the behaviour of functional signalling complexes within the cellular environment: as these proteins contain domains prone to self-aggregation, they can oligomerise into long fibrils when overexpressed, potentially limiting the relevance of these findings to native complex assembly at endogenous expression levels.

This limitation has begun to be addressed through the development of fluorescently labelled cell models expressing physiological concentrations of MyD88 and TRIF, allowing for the visualisation of the signalling-competent SMOCs which direct TLR activation: the MyDDosome and TRIFosome [10]. A further advance has been the development of single-molecule pulldown (SiMPull), which immobilises individual complexes from cell lysate on a microscope slide via an antibody against one component, allowing the fluorescently labelled proteins within each complex to be counted individually rather than averaged across the population [11]. Although the biochemical effectors of the TRIF pathway are well catalogued, predominantly for the expression of interferons, and fluorescence imaging and SiMPull studies have proved vital in demonstrating the real-time formation of signalling complexes, our understanding of the structural architecture of the endogenous TRIFosome and its functional consequences at physiological concentrations of adaptor proteins remains sparse. Understanding how and why TRIF oligomerises into a heterogeneous mix of scaffold sizes and protein associations will be important in furthering our understanding of how TRIF can direct different cellular responses to PAMPs, spanning inflammatory signalling, interferon signalling, and the initiation of cell death pathways.

TRIF’s multifunctional role in signalling can be attributed to its underlying domain architecture, which can be separated into four regions, each central to overall signalling function. TRIF contains an N-terminal domain, separated from a TIR domain by an unstructured region. This region contains interaction sites for TNF receptor-associated factor 6 (TRAF6), which allow for the initiation of downstream signalling through the transcription factor NF-κB, as well as a pLxIS sequence, which plays a role in interferon regulatory factor 3 (IRF3) signalling following phosphorylation by TANK-binding kinase 1 (TBK1) [12,13]. The TIR domain enables homotypic interactions with the upstream TLRs, either directly in the case of TLR3 signalling, or via the TIR domain of the adaptor protein TRAM in the case of TLR4 signalling [14]. Whilst all TLRs can utilise their TIR domain to interact with downstream adaptors, only TLR3 and TLR4 have the suitable surface-residue configurations and BB-loop structures in order to recruit TRIF directly, or via the bridging protein TRAM [15]. Finally, the C-terminal region contains a RIP homotypic interaction motif (RHIM), which enables the recruitment of RIPK1 to TRIF, driving a secondary TIR-independent route for NF-κB activation, alongside enabling RIPK1-, FADD-, and caspase-8-dependent apoptosis [16]. The RHIM also plays a second role in cell death via the direct association of RIPK3; when caspase-8 activity is switched off, RIPK3 association can drive MLKL-dependent necroptosis [17]. Collectively, these domains enable TRIF’s role as a signalling hub, in which specific protein interactions—TBK1/TRAF-dependent signalling via the N-terminal domain, or RIPK1/RIPK3-dependent signalling via the RHIM—determine functional output. Whether this modularity is mirrored at the level of individual endogenous complexes and how the structural heterogeneity of the TRIFosome in turn shapes which output dominates is considered below.

## What determines TRIFosome structural heterogeneity?

Recent *in situ* imaging reveals that endogenous TRIFosomes exhibit pronounced heterogeneity in both size and stoichiometry following TLR3 or TLR4 activation [18]. This contrasts with the architecture of other characterised SMOCs; for example, the inflammasome typically nucleates a single macromolecular aggregate (the ASC speck) per cell [19,20]. In contrast, cells frequently assemble multiple, morphologically distinct TRIF complexes simultaneously, suggesting that TRIFosome assembly follows variable kinetics. This structural heterogeneity may not be arbitrary, but rather a functional prerequisite that enables a single adaptor to coordinate TRIF’s multidirectional signalling profile.

The biological mechanisms governing this structural diversity remain a critical unresolved question. A purely temporal model suggests that the observed heterogeneity represents distinct kinetic snapshots of a uniform assembly pathway following initial seeding of the TRIFosome at a TLR; in this scenario, all TRIFosomes follow identical growth kinetics, and variations in the size of complexes are dictated solely by the time elapsed since initial nucleation at the receptor. However, this proposed hypothesis does not explain why mature TRIFosomes are also capable of forming a wide morphological distribution. Single-molecule fluorescence imaging of endogenously labelled TRIFosomes revealed that only a subset of complexes adopts an elongated, non-circular morphology approaching one micron [18]. An alternate hypothesis is that the morphological fate of individual complexes is determined by the interplay between TIR and RHIM domain interactions, potentially favouring widespread RHIM cross-linking in some complexes, whilst restricting others to smaller, TIR-dominant architectures. RHIM domains nucleate amyloid-like filaments *in vitro*, a mechanism first characterised for RIPK1/RIPK3 in the necroptosome [21]. These amyloid fibrils are characteristically stable and resistant, potentially forming the protease-resistant core identified following subtilisin treatment of recombinant full-length TRIF *in vitro* [18].

How TRIF’s domains give rise to structurally distinct assemblies has been studied through structure-function studies of the recombinant protein *in vitro*. Whilst expression of TRIF’s TIR domain alone is sufficient to nucleate filament formation [9,22], introducing a mutation in the RHIM core tetrad of recombinant full-length TRIF, thereby limiting RHIM–RHIM interactions, formed shorter filaments compared with WT TRIF [18], demonstrating that homotypic RHIM interactions make a distinct contribution to TRIF filament growth beyond TIR-mediated oligomerisation alone. Whether this structural distinction between TIR- and RHIM-mediated assembly is reflected in signalling outcome remains to be established; single-molecule pulldown analysis of endogenous TRIFosomes offers one way to begin addressing this. Single-complex pulldown analysis of endogenously labelled TRIFosomes revealed that RIPK1 preferentially associates with the ‘mature’, brightest TRIF complexes, whilst phospho-TBK1 accumulates prior to the assembly of these stable complexes, suggesting that early TBK1 association with TRIF may be initiated from lower-order, more transient TRIF assemblies [18]. Together, these findings suggest that the growth of TRIF assemblies from lower-order, potentially TIR-dominated assemblies towards larger, RHIM-cross-linked mature complexes may structurally separate early-stage interferon signalling via TBK1 and inflammatory signalling via RIPK1 (Figure 1). RHIM engagement might therefore act as a structural switch, determining not only the size and stability of the resulting TRIFosome, but also the specific signalling pathway a complex ultimately directs. The stability of the ‘mature’ TRIFosome may also arise from RHIM-mediated filament branching, in which TRIF incorporated into a RHIM-cross-linked filament retains free TIR domains capable of nucleating further TIR-mediated oligomerisation, generating an interconnected network (Figure 2). Such an architecture has not been directly resolved and would require structural characterisation of the endogenous complex to test; however, branching has been observed in other SMOCs such as ASC complexes [23]. In the case of NLRP3-activated ASC complexes, which are hypothesised to cross-link through CARD–CARD interactions, branching likely stabilises the assembly whilst leaving binding sites freely accessible throughout the network, allowing the speck to serve as a platform for caspase-1 recruitment and activation, driving IL-1β maturation and pyroptosis [23]. Phospho-TBK1 is also not necessarily restricted to associating with early TRIF complexes, but may persist alongside RIPK1 as the scaffold matures, potentially consistent with its already established role in limiting RIPK1-dependent cell death within the TNF receptor 1 (TNFR1) signalling complex [24]. Introducing this RHIM mutation into endogenous cell models would help test these theories directly; SiMPull techniques could then be used to study RIPK1 associations with RHIM-mutated TRIFosomes, whilst fluorescence recovery after photobleaching (FRAP) techniques would allow for further investigation into TRIFosome complex stability following disruption of RHIM–RHIM interactions.

**Figure 1:**
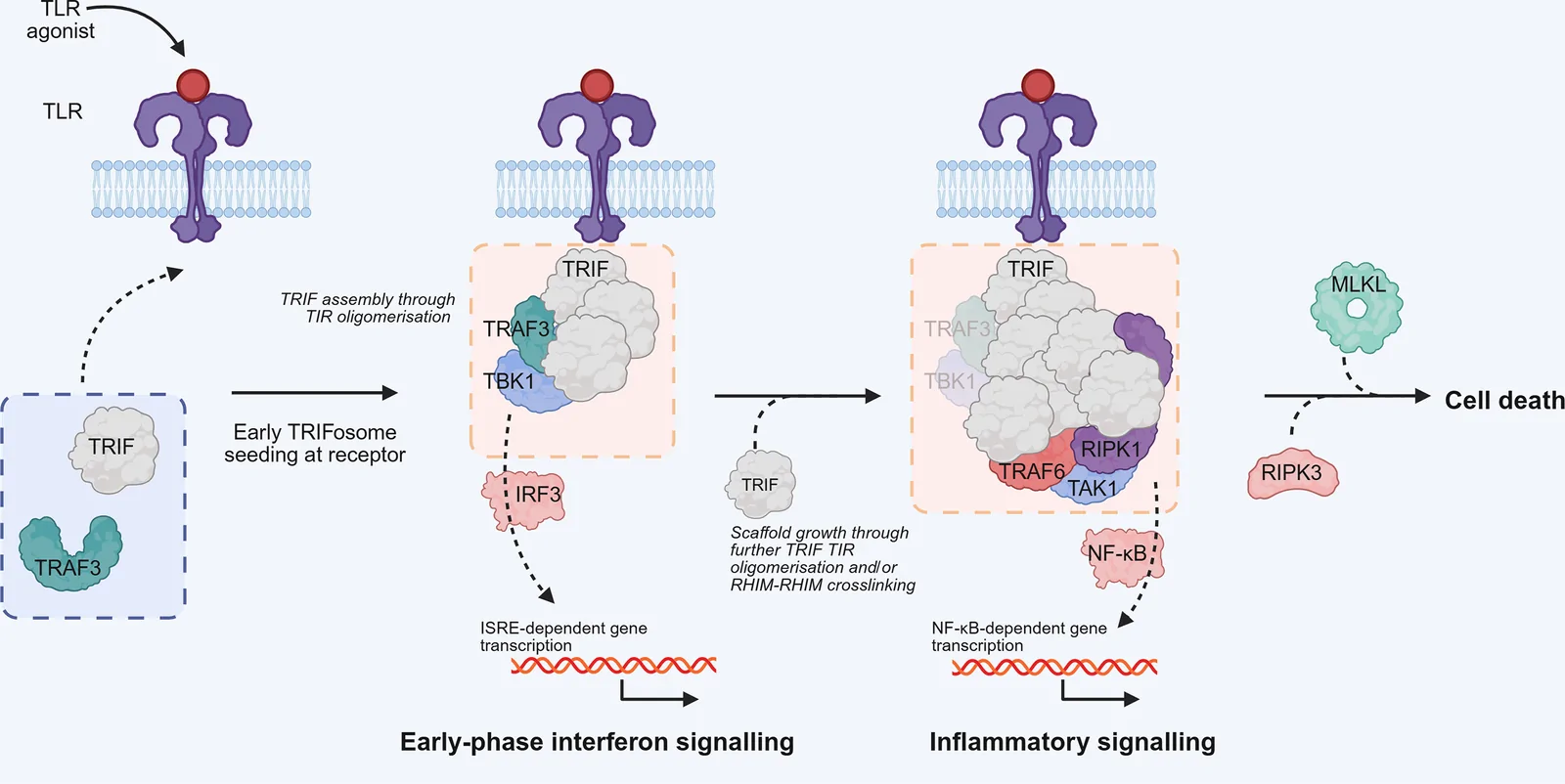
Proposed modular signalling from distinct TRIF assemblies Following TLR activation, the early TRIF assembly is nucleated through TIR oligomerisation, recruiting TBK1 to drive early-phase interferon signalling through IRF3. TRIF scaffolds mature and stabilise through further TIR oligomerisation and RHIM–RHIM cross-linking, recruiting RIPK1 to drive inflammatory signalling through NF-κB. Upon loss of caspase-8 activity, RIPK3 is recruited to mature, RHIM-cross-linked scaffolds, driving MLKL-dependent necroptotic cell death; the precise route of RIPK3 engagement remains undetermined. Created using BioRender.

**Figure 2:**
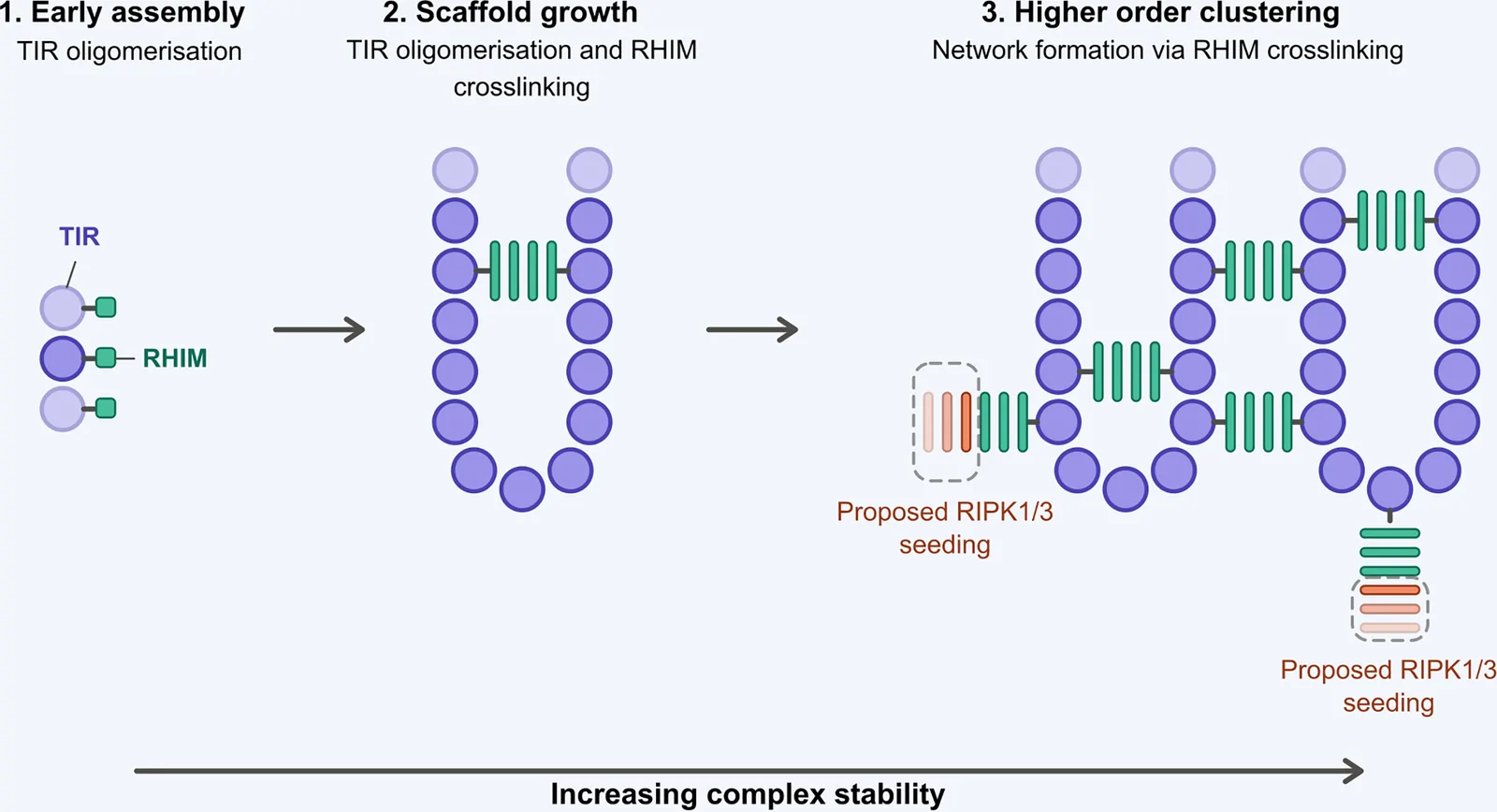
Proposed schematic for TRIF complex assembly 1) TRIF nucleates a transient complex through TIR oligomerisation. 2) Further filament growth through TIR oligomerisation is stabilised through RHIM–RHIM interactions within the growing assembly. 3) RHIM cross-linking between filaments generates more stable higher-order assemblies. RHIM–RHIM interactions can seed the formation of RIPK1/RIPK3 amyloid filaments. Created using Inkscape.

Our understanding of the associations between the endogenous TRIFosome and cell death remains an open question. Similar to the differences hypothesised between ‘transient’ and ‘mature’ TRIFosomes in directing interferon and inflammatory signalling, does a small subset of total TRIFosome assemblies in each cell coordinate downstream cell death responses? TRIF-dependent complexes containing RIPK1, FADD, and caspase-8 can assemble and drive cell death in response to LPS and *Yersinia pseudotuberculosis*, initiated through ZBP1-RIPK1 binding [25], and can also form downstream of TNFR1 [26]. However, ensemble biochemical approaches are unable to determine if these complexes represent a distinct subpopulation of TRIF assemblies. In cellular models, RIPK3 engagement with TRIF’s RHIM is specifically linked to compromised caspase-8 activity [17], which raises the question of whether RIPK3 can associate with an already RIPK1-associated TRIFosome, or whether it requires its own distinct route of assembly. RIPK1–RHIM associations with TRIF may, like the RIPK1–RIPK3 necroptosome, begin with an initial nucleation event, followed by further sequential association into a stable structure [21]. This process occurs within 45–60 min following TLR4 activation, when the mature, RIPK1-associated TRIFosome has fully formed [18]. If a similar process occurs between TRIF and the RIP kinases, RIPK3 may not be recruited to the assembly until RIPK1 has already seeded the RHIM–RHIM interaction, though this has not yet been demonstrated for the endogenous TRIFosome. An alternate possibility is that RIPK3 does not engage with TRIF’s RHIM directly, instead associating with the RHIM of RIPK1, extending the protein complex in the alternating RIPK1/RIPK3 arrangement that is characteristic of the canonical necroptosome [21]. Resolving which of these models holds true, through the use of time-resolved single-complex pulldowns and cross-linking mass spectrometry, will be important for understanding how TRIF directs cell death and determining whether this branch of TRIF signalling could be targeted by therapeutics independently of its early interferon and inflammatory outputs.

## What constrains TRIFosome size?

Single-molecule fluorescence imaging of the TRIFosome reveals an unexpected feature of its assembly. Despite the comparatively low endogenous expression of TRIF, TLR activation does not deplete the monomeric pool, with a substantial fraction of unassembled TRIF persisting even in cells that form a large number of mature complexes [18]. This suggests that, unlike the filaments formed following recombinant expression of TRIF that can extend to several microns in length, TRIFosome size within the cellular environment is likely capped [9]. This may be a mechanism within the cell to prevent runaway signalling as seen, for example, when overexpression of adaptor proteins such as MyD88 leads to large artefactual complexes and its overactivation [27]. Determining what constrains TRIFosome size is likely to be of functional consequence, rather than purely a structural detail. Within the analogous MyDDosome, for example, MyD88 oligomer size has been shown to act as a threshold for activating downstream signalling in cells endogenously expressing fluorescently labelled MyD88 [28]. One possibility is that the recruitment of downstream adaptors may play a role in imposing a limit on scaffold growth. This is an established phenomenon in MyDDosome oligomerisation, where, in the same endogenous system, the absence of the kinase IRAK4 results in the formation of larger MyD88 assemblies [28]. If RIPK1 can play a similar role within the TRIFosome, its recruitment might similarly limit RHIM-mediated protein complex growth. Notably, however, this ‘capping’ behaviour is not a universal feature across death-fold domain scaffolds and their downstream effectors. In the inflammasome, ASC is reported to be rapidly and almost completely depleted from the monomeric pool to form the large ASC speck post-activation, despite caspase-1 recruitment occurring via CARD–CARD interactions during assembly of the speck [29]. This uncapped mode of complex growth might offer a mechanistic basis for the single, more uniformly sized aggregates that the inflammasome forms within the cell, in contrast with the more heterogeneous and numerous TRIFosome (and MyDDosome) assemblies that form within a single cell. Effector recruitment is therefore unlikely to be sufficient alone to explain the constraints on TRIFosome scaffold growth. A further possibility is that the low endogenous concentration of TRIF is itself limiting. Since TRIF is monomeric at rest and requires receptor-proximal nucleation to assemble, the number of complexes formed is set by the number of engaged receptors rather than by TRIF abundance, so that a limited pool of protein is partitioned across many simultaneously nucleating assemblies. Under such a model, no individual complex could reach the lengths achieved by recombinant TRIF *in vitro*, and no active mechanism would be required to terminate growth. A third possibility is that the affinity of TRIF–TRIF interactions within the complex is low, such that assembly is reversible, with a substantial monomeric fraction persisting in equilibrium with the assembled complexes. This hypothesis, however, would be difficult to reconcile with the stability of the TRIF complexes, which can be recovered intact following detergent-based lysis [18], conditions under which transient interactions might be expected to dissociate. This could be directly investigated in endogenous cell models using FRAP, whilst the development of RIP kinase knock-out cell models will likely prove critical in understanding the contribution of effector recruitment to scaffold growth.

## Translational opportunities

Across the study of innate immune signalling complexes, overexpression and recombinant protein studies have proved valuable in identifying plausible assembly mechanisms; however, further advancing our understanding of complex architecture will increasingly rely on the development of endogenous models. These tools are enabling better understanding of how the scaffold function of aggregate-prone adaptor proteins, such as TRIF, contributes to different signalling outcomes, with direct consequences for how therapeutics against infection and chronic inflammatory disease are designed. Alongside the development of therapeutics targeting upstream innate immune receptors (E5564 (eritoran) and TAK-242 (resatorvid) for TLR4) or downstream cytokines (such as IL-1β inhibitory biologics), another compelling target involves directly targeting the oligomerising adaptor protein involved in scaffold assembly. Such an approach is currently in pre-clinical testing for MyD88 [30]. The TRIFosome might be particularly well suited for this strategy. Unlike the MyDDosome scaffold, which is hypothesised to drive predominantly NF-κB-driven inflammatory signalling, distinct TRIFosome complexes, distinguished through the size of the TRIF assembly and potentially also the degree of RHIM oligomerisation, appear to differentially direct interferon and inflammatory signalling. Selectively disrupting RHIM-mediated interactions, rather than targeting TRIF as a whole, could potentially suppress the inflammatory output driven through mature, RHIM-cross-linked assemblies whilst leaving TIR-mediated interferon signalling unaffected. These strategies could unlock a distinct mode for restricting chronic inflammation in disease, whilst still allowing the antiviral production of type I interferons.

This logic could also extend to the development of RIPK1 inhibitors, a key therapeutic target in neurodegenerative disease and autoimmunity, given its role in cell death and inflammation [31]. Development has so far been focused on inhibitors that bind RIPK1’s kinase domain, with few, if any, currently targeting the RHIM interface through which RIPK1 can scaffold with RIPK3 in the necroptosome or TRIF in the TRIFosome. Translation of these compounds from mouse models to human systems has also been limited by species differences in RIPK1 activation [32]. Critically, these inhibitors do not affect RIPK1’s scaffold function, potentially preserving TRIF- and RIPK3-containing scaffolds even when RIPK1’s catalytic activity is inhibited. Targeting both RIPK1’s catalytic and scaffolding functions simultaneously may therefore prove to be a more successful approach in therapeutic development than kinase inhibition alone. These ideas have already been applied in the development of RIPK1 PROTACs, which deplete RIPK1 entirely, removing both its catalytic and scaffolding functions [33]. In cell models, acute RIPK1 depletion enhanced TLR3/4-induced necroptosis and separately protected against RIPK1-driven skin inflammation *in vivo*, in contrast with kinase inhibitors, which failed to suppress RIPK1-induced apoptosis in human cell lines [33]. This also mirrors the strategy that has been used for IRAK4, which has a similar catalytic and scaffolding role in the MyDDosome [34]. The limited efficacy of kinase inhibitors motivated the development of IRAK4 degraders, which have shown improved suppression of downstream cytokine outputs compared with kinase inhibitors alone [35]. Unlike degradation, selectively targeting the TRIF–RIPK1 RHIM interface could instead retain pathway selectivity. RIPK3 engages through RHIM interactions, whilst caspase-8 is recruited via FADD and RIPK1’s death domain, so a RHIM-selective agent would spare apoptotic signalling at receptor complexes that recruit RIPK1 through its death domain, which whole-protein depletion would remove.

Beyond therapeutic intervention, the same structural heterogeneity that defines TRIFosome assembly may also hold diagnostic value. Single-molecule studies of innate immune complexes suggest they may represent powerful translational targets, as their physical structures preserve rich biological detail. Individual immune complexes carry a multidimensional array of information in their abundance, morphology, molecular composition, and post-translational modifications. This makes higher-order assemblies fundamentally different from conventional biomarkers; their heterogeneity contains information that ensemble assays tend to average away. A similar way of thinking has already been applied to ASC specks as a biomarker, where measuring ASC specks from biofluids such as blood plasma, serum, and cerebrospinal fluid has revealed differences in abundance and morphology between healthy controls and patients with Alzheimer’s disease, Parkinson’s disease and myelodysplastic syndromes [36,37]. A similar premise could be applied to TRIFosomes; given the wide heterogeneity of complexes highlighted in this review, the main opportunity lies in moving away from determining the abundance of TRIFosomes towards mapping their molecular identity in biological assays. A ‘next-generation’ diagnostic assay, looking for disease-linked changes in protein interactions or post-translational modifications, could transform a generic readout of immune activity into a mechanistically informative ‘molecular fingerprint’.

Looking ahead towards future translational opportunities, diagnostics and therapeutics thus converge on a single molecular recognition challenge in targeting specific TRIFosome assembly states. A binder that is selective for a specific disease-associated assembly can first isolate and classify an intact TRIF assembly and subsequently act as a therapeutic if the assembly is pathogenic [38]. The key translational bottleneck therefore lies not purely in assay sensitivity, but in identifying and isolating intact, heterogeneous complexes. Solving this has the potential to transform TRIFosomes from bulk markers of innate immune signalling into mechanistically resolved biomarkers and assembly-selective therapeutic targets.

## Future directions

The combined picture emerging from structural studies of purified TRIF, as well as endogenous studies of the functional TRIFosome within cells, is beginning to reveal a mechanism wherein TRIF can coordinate distinct outputs through heterogeneous TRIF assemblies, distinguished by their size and effector composition. The generation of endogenously expressed TRIF mutants in cell models which alter TIR/RHIM oligomerisation will allow for a more precise understanding of how scaffold architecture directs output, guiding the development of novel therapeutics which can shift the balance between TRIF’s inflammatory, interferon and cell death signalling, rather than uniformly suppressing all downstream pathways. A key theme across innate immune adaptor proteins is their markedly different structural behaviour in overexpression versus endogenous cell models. Direct visualisation of endogenous TRIFosome architecture within cells, for instance via cryo-electron tomography, would resolve the structure-function relationship between TRIF and its recruited kinases without relying on comparison to purified or overexpressed systems, and could additionally reveal how complex architecture evolves kinetically following receptor activation. Furthermore, the role of the endogenous TRIFosome in directing cell death is of particular interest, given the relevance of RIPK1/RIPK3-driven necroptosis in driving neuronal cell death in neurodegenerative conditions such as Alzheimer’s disease. A further direction for study concerns TRIF’s domain architecture itself. The *Lps2* allele, a frameshift in *Ticam1*, phenocopies Ticam1 knockout mice, and has been used accordingly across much of the literature, although it removes only a short C-terminal segment lying outside TRIF’s characterised interaction domains [7]. That such a small change abolishes TRIF signalling suggests that regions outside the TIR and RHIM domains contribute to function in ways not captured by current models, adding a further layer of complexity to understanding TRIF signalling.

It is also tempting to speculate that the proposed clustering mechanism could address evolutionary pressure. In complex II, for example, RIPK1 may be the most evolutionarily conserved gene, followed by caspase-8, with FADD potentially having evolved later to bridge and balance the two. A phylogenetic analysis would be required to explore the validity of this hypothesis.

A key experimental theme that will further our understanding of TRIF’s mechanistic role will be the development of new tools that allow for the identification and isolation of intact TRIF assemblies, allowing for better mapping of TRIFosome heterogeneity. By extending single-molecule imaging approaches like SiMPull across a panel of TRIF binding partners, alongside the development of single-complex proteomic methods to more extensively characterise the protein interactions within distinct TRIFosome populations, it will become possible to resolve directly how disease-associated TRIF variants disrupt complex assembly, connecting the structural principles established in this review to human disease.

## Perspectives

- TRIF-dependent signalling sits at the intersection of antiviral immunity, chronic inflammation, and cell death. Understanding how TRIF can coordinate its diverse downstream outputs has direct consequences for the development of therapeutics that can treat neurodegenerative and autoimmune diseases.
- Imaging of endogenous TRIFosomes in cells reveals that TRIF assembles into structurally heterogeneous complexes, with smaller, transient complexes directing early interferon signalling, and larger, mature complexes driving inflammatory signalling via RIPK1. These functional differences may be explained by distinct modes of TRIF oligomerisation, with TIR-domain interactions nucleating smaller assemblies and RHIM-mediated cross-linking driving growth into larger, stable complexes.
- Future studies, utilising advances in single-molecule pulldown techniques to isolate intact complexes, can work towards mapping the composition and architecture as a ‘molecular fingerprint’ for each assembly. Further understanding of how TRIF scaffold composition and architecture direct interferon, inflammatory, and cell death outputs will inform the development of novel therapeutics capable of selectively biasing TRIF signalling in disease.

## Open Access

Open access for this article was enabled by the participation of the University of Cambridge in an all-inclusive Read & Publish agreement with Portland Press and the Biochemical Society under an agreement with JISC.

## Abbreviations

ASC: apoptosis-associated speck-like protein containing a CARD
CARD: caspase recruitment domain
FADD: Fas-associated death domain
FRAP: fluorescence recovery after photobleaching
IL-1β: interleukin-1β
IRAK4: interleukin-1 receptor-associated kinase 4
IRF3: interferon regulatory factor 3
MLKL: mixed lineage kinase domain-like pseudokinase
MyD88: myeloid differentiation primary response protein 88
NF-κB: nuclear factor kappa B
NLRP3: NLR family pyrin domain-containing 3
PAMPs: pathogen-associated molecular patterns
PROTAC: proteolysis-targeting chimera
RHIM: RIP homotypic interaction motif
RIPK1: receptor-interacting serine/threonine protein kinase 1
RIPK3: receptor-interacting serine/threonine protein kinase 3
SiMPull: single-molecule pulldown
SMOCs: supramolecular organising centres
TBK1: TANK-binding kinase 1
TIR: Toll/interleukin-1 receptor/resistance protein
TLR: Toll-like receptor
TNFR1: TNF receptor 1
TRAF6: TNF receptor-associated factor 6
TRAM: TRIF-related adaptor molecule
TRIF: TIR domain-containing adaptor inducing interferon-β
ZBP1: Z-DNA binding protein 1
